# Deficits in ascending pain modulation pathways in breast cancer survivors with chronic neuropathic pain: A resting-state fMRI study

**DOI:** 10.3389/fneur.2022.959122

**Published:** 2022-12-08

**Authors:** Rui Liu, Na Qiao, Shuwei Shi, Suyao Li, Yingman Wang, Jie Song, Wenting Jia

**Affiliations:** ^1^Department of Chemoradiotherapy, North China University of Science and Technology Affiliated Hospital, Tangshan, Hebei, China; ^2^Department of Breast Surgery, The First Hospital of Qiqihar, Qiqihar, Heilongjiang, China; ^3^Department of Breast Surgery, Affiliated Qiqihar Hospital, Southern Medical University, Qiqihar, Heilongjiang, China

**Keywords:** breast cancer, functional MRI, chronic neuropathic pain (CNP), depression, functional connectivity (FC)

## Abstract

**Purpose:**

Breast cancer (BC) is the highest frequent malignancy in women globally. Approximately 25–60% of BC patients with chronic neuropathic pain (CNP) result from advances in treating BC. Since the CNP mechanism is unclear, the various treatment methods for CNP are limited. We aimed to explore the brain alternations in BC patients with CNP and the relationship between depression and CNP utilizing resting-state functional magnetic resonance imaging (rs-fMRI).

**Methods:**

To collect the data, the female BC survivors with CNP (*n* = 20) and healthy controls (*n* = 20) underwent rs-fMRI. We calculated and compared the functional connectivity (FC) between the two groups using the thalamus and periaqueductal gray (PAG) as seed regions.

**Results:**

Patients with BC showed increased depression and FC between the thalamus and primary somatosensory cortices (SI). Moreover, the Hospital Anxiety and Depression Scale-Depression (HADS-D) and pain duration were linked positively to the strength of FC from the thalamus to the SI. Furthermore, the thalamus-SI FC mediated the impact of pain duration on HADS-D.

**Conclusion:**

In BC patients with CNP, the ascending pain regulation mechanism is impaired and strongly associated with chronic pain and accompanying depression. This research increased our knowledge of the pathophysiology of CNP in patients with BC, which will aid in determining the optimal therapeutic strategy for those patients.

## Introduction

Breast cancer (BC) is the highest prevalent malignancy affecting women globally (1). With increased treatment efficacy (surgery, radiotherapy, hormonal treatment, chemotherapy, or combined treatment), this disease has more survivors than ever (2). However, many survivors suffer from chronic pain, including chronic neuropathic pain (CNP), with approximately 25–60% of women with CNP after receiving BC medication (3). CNP is a collection of distinct chronic pain manifestations induced by harm or disorder of the somatic sensory system, such as post-surgical nerve and tissue damage and inflammation exceeding 3 months. It is a syndrome caused by nerve dysfunction due to extensive nerve fiber injury (4, 5). Symptoms include all kinds of debilitating pain, including burning, shooting, and stabbing, which usually lasts indefinitely (4, 6). Accordingly, the mechanism of the CNP is still unclear, and the symptoms are more resistant than other types of pain to available treatments (7). Therefore, patients frequently experience complications for the remainder of their lives. Depression, observed in 18–54.4% of patients with BC, is among the highest prominent psychological conditions (8–11). Depression seriously affects mental health, work, and life (12). Meaningfully, depression is related to elevated deaths in patients with BC (13). Depression is the primary factor affecting the functional status of patients with BC (14). A good correlation exists between pain and depression in patients with BC (15, 16). Although this is a common phenomenon, its brain mechanism remains unclear.

Functional MRI (fMRI) is a commonly recognized method to examine brain function, especially in pain research. CNP involves morphological changes and functional adaptations of brain processing (17, 18). Damage to the ascending and descending pain regulation mechanisms may account for the aberrant sensory manifestations of people with chronic pain (19). The thalamus can transmit nociceptive signals to the somatosensory cortices (SI) through the spinothalamic projection, which is crucial in the ascending pain pathway (20). The descending pain regulation mechanisms are significantly involved in pain perception. These pathways project to neurons in the dorsal horn of the spinal cord to control the ascending information of pain, and the higher cortex dominates the periaqueductal gray (PAG), which is the main control center in the descending pain regulation pathway (21). Li et al. (22) discovered aberrant functional connectivity (FC) patterns across ascending and descending pain mechanisms in individuals with post-herpetic neuralgia, where the thalamus and PAG were utilized as seeds. In numerous chronic pain cases, abnormal FC of the pain regulation system has been reported, such as migraines (23), low back pain (24), painful diabetic neuropathy (25), chronic neuropathic pain (17), trigeminal neuralgia (26), sciatica (27), and neuropathic pain (28). Nevertheless, it is unclear how the ascending and descending pain regulation systems lead to CNP in patients with BC. Moreover, the connection between chronic pain and depression in patients with BC remains unexplored.

Herein, we utilized fMRI to evaluate neural changes in ascending and descending pain mechanisms in BC patients with CNP and to assess the link between CNP and depression in depth. We speculated that the FC between the thalamus, PAG, and other brain areas in BC patients with CNP was disrupted and that a defective FC may be associated with pain features and depression. If the hypotheses are confirmed, our study will enhance the understanding of the CNP in BC, which will greatly benefit the development of new treatments for BC.

## Methods

### Subjects

Our research complied with the recommendations of the Declaration of Helsinki, and the Local Ethical Institutional Review Board accepted the procedures. Before each procedure, all subjects signed informed consent. This research involved 20 right-handed patients with BC (20 women: mean age 52.25I ± 6.55 years) and 20 ideally suited healthy right-handed controls (20 women: mean age 49.95 ± 6.06 years).

The inclusion criteria included (1) patients with BC survived 1-year or more post-therapy and experienced CNP for at minimum 3 months, (2) 18 years of age or older, and (3) a pain rating equal to or exceeding 3 out of 10. Exclusion criteria included (1) cognitive decline; (2) brain metastases; (3) past psychiatric disorders; (4) receiving central pain treatment throughout 1 month; (5) concurrent illnesses, like severe infection or systemic illness (rheumatologic, cardiac, respiratory, gastrointestinal, neurological, and endocrine); (6) past events of neural disorders or dementia, psychiatric illnesses, or failure to accomplish the test methods and MRI scans; and (7) an unwillingness to participate in the study.

### Demographic and clinical features

At admission, demographics (marital status and education) and clinical data (cancer stage, surgeries and therapies administered, and antidepressant and/or pain killers use) were acquired through self-reports and electronic health records.

### Pain

Peripheral painkillers were stopped 1 week before the fMRI scan, and no painkillers were given during the scanning to ensure the accuracy of the data. We recorded the duration of CNP for each patient as pain duration. The CNP of patients with BC was evaluated using the visual analog scale (VAS) score (0–10 cm, with greater scores showing increased pain). Subjects indicated their current and historical pain levels by pointing to a spot on the line between the two faces. One experienced pain specialist measured the VAS (past) and VAS (during scanning) of patients to evaluate the pain intensity.

### Depression

The hospital anxiety and depression scale (HADS) is a 14-item self-reported survey with anxiety and depression subscale scores (29). HADS performs well in evaluating anxiety disorders and depression symptoms in patients with BC (29–32). We assessed the incidence and degree of depressive manifestations using the hospital anxiety and depression scale-depression (HADS-D). Each HADS-D item varies from 0 (no symptoms) to 3 (severe symptoms), while depression total scores are calculated as aggregates varying from 0 to 21. The reference range is 0–7, 8–10 indicates the status, and 11 or more indicates depression. The HADS-D has demonstrated reliability and validity for depression in different demographics, including patients with BC. The measure requires around 3 min to complete. We measure all subjects using HADS-D.

### Statistical analysis

Using two-sample *t*-tests, age and educational years variations between patients with BC and healthy controls were determined. A Chi-square test was utilized to examine gender disparity between the two groups.

### fMRI data acquisition and preprocessing

A Philips Ingenia 3T MR scanner (RoyalPhilips, Amsterdam, the Netherlands) was employed to obtain 3T fMRI results. Subjects were asked to maintain their heads steady throughout scanning, and a sponge pad was utilized to limit unconscious head motion. Also, subjects were instructed to stay awake and close their eyes, and refrain from engaging in specific or intense thinking.

BOLD signals were acquired utilizing a gradient-echo-planar imaging sequence (EPI) with the next metrics: TR (repetition time) = 2,000 ms, TE (echo time) = 30 ms, flip angle = 90°, slices = 36, slice thickness = 4 mm, slice spacing = 4 mm, matrix = 128 × 128, volumes = 200, volume interval = 2 s, and voxel size = 2 × 2 × 2 mm^3^. Concurrently, high-resolution T1-weighted structural photos were acquired using a 3-dimensional magnetization prepared rapid acquisition gradient echo sequence (MPRAGE) with the next scanner metrics: TR = 7.44 ms, TE = 3.46 ms, flip angle = 8°, sagittal slices = 301, slice thickness = 1.2 mm, slice spacing = 0.6 mm, image matrix = 240 × 240, volume = 1, and voxel size = 1 × 1 × 1 mm^3^.

A brief description of preprocessing pipeline was as follows: (1) the initial 10 volumes of each functional scan matching every subject acclimation to the scanning setting and magnetization stabilization were neglected; (2) movement adjustment was conducted to lessen the impacts of head movement; and (3) functional photos were co-registered to structural photos and spatially normalized to the Montreal Neurological Institute template. Each voxel was resampled to 3 × 3 × 3 mm^3^; (4) the liner-drift, Friston-24 criteria, the white matter signal, and the CSF signal were retrieved as covariates and regressed to lessen non-neural signals; (5) a scrubbing action was also conducted for high motion time points; and (6) eventually, a band pass filter (0.01–0.08 Hz) was used to eliminate high-frequency noise influences and smoothed with an 8 mm full-width-half-maximum isotropic Gaussian kernel. Therefore, the collected finding was analyzed deeper.

Considering that the thalamus and PAG are important nodes in the ascending and descending pain mechanisms, they were selected as the starting points for the FC analysis of resting-state fMRI (rs-fMRI) data. To ensure homogeneity among all participants following standard fMRI analysis pipelines, the thalamus seed was derived from the Harvard Oxford subcortical structural atlas (90% threshold), a population-based probability atlas in MNI-152 standard space (33). The PAG seed was determined using the Duvernoy atlas of the Human Brainstem and Cerebellum in MNI-152 standard space (34). Resting-state seed-based FC analysis was conducted utilizing the toolbox Data Processing Assistant for rs-fMRI (DPARSF; http://www.restfmri.net/forum/DPARSF) pipeline.

The mean time series of the thalamus and PAG were extracted, and voxel-wise FC was calculated between the thalamus and the remaining voxels within the brain. The same method was used to calculate the FC of PAG. The differences in the seed-based FC in patients with BC and HC were assessed using a general linear model within the gray matter masks. The grouping information was set as contrast while age, gender, and educational years were set as covariables to regress their linear effect on FC. *p* ≤ 0.001 was corrected for multiple comparisons using familywise error correction at the cluster level, matching to an adjusted *p* ≤ 0.05, using statistical parametric mapping (SPM12; http://www.fil.ion.ucl.ac.uk/spm). Subsequently, the mean value within the resultant cluster was extracted and linked to the clinical indicators in patients with BC.

### Mediation analysis

A bootstrapped mediation analysis was employed to evaluate the mediatory link among pain duration, HADS-D, and seed-based FC. Using 2000 bootstrap samples and the PROCESS macro (http://processmacro.org, vs. 2.16.3) in statistical product service solutions (SPSS; IBM, vs. 23.0), 95% confidence intervals (CI) for model parameters were determined. The objective of the mediation analysis was to examine if a significant variation existed between the total effect (path c) and the direct effect (path c′) that the mediator (M) accounts for. Two models were evaluated using the thalamus-SI FC as the mediator: (1) pain duration to be the independent variable and HADS-D ratings to be the dependent variable and (2) HADS-D ratings to be the independent variable and pain duration to be the dependent variable. A mediation was considered significant when bootstrapped upper as well as lower 95% CIs showed a number except zero.

### Validation analysis

To examine the consistency of the present findings. We also regressed the global signal in our current data and performed FC, correlation, and mediation analyses identical to our previous analyses.

## Results

### Demographics, pain, and depression features

**Figure 1:**
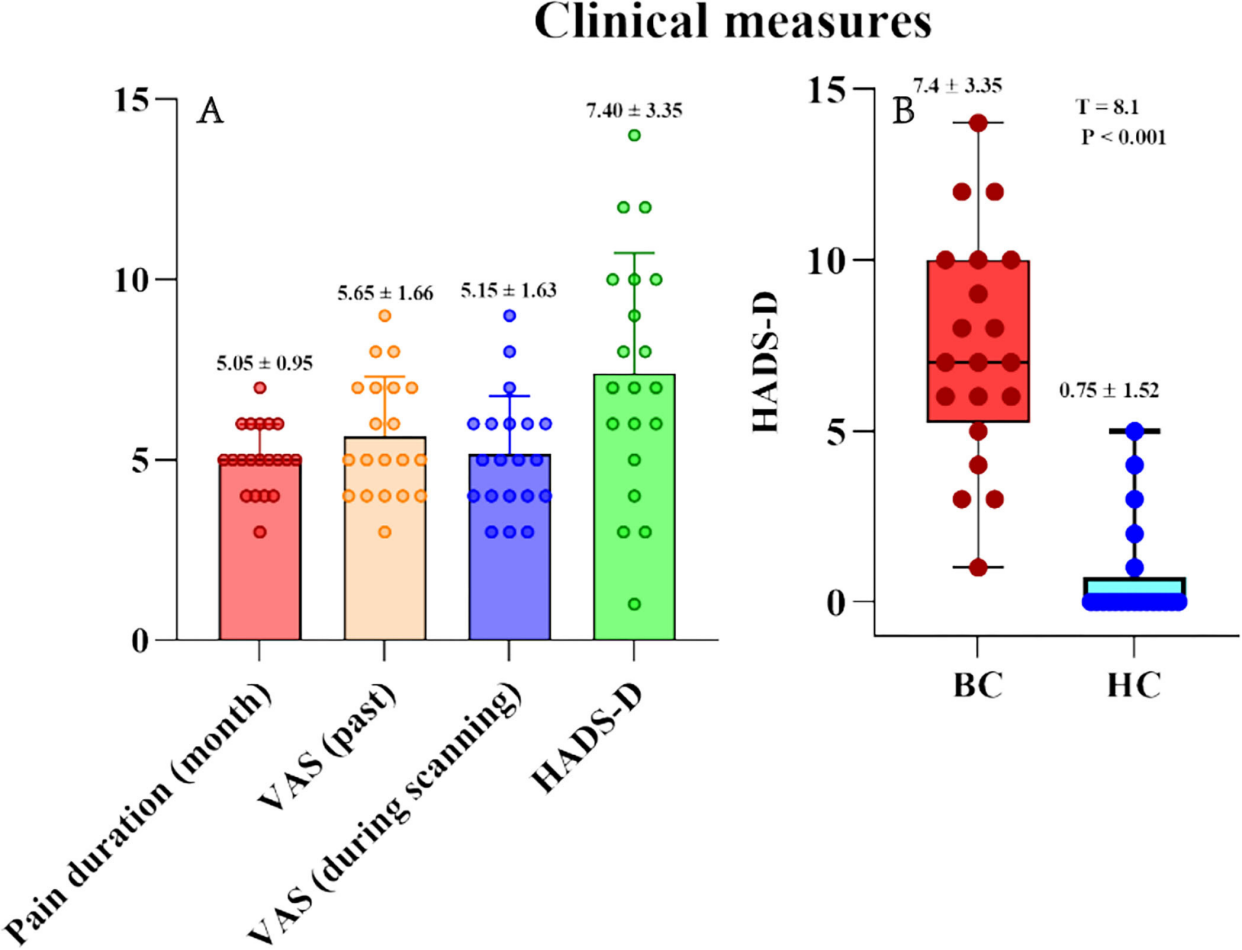
Pain characteristics (VAS and pain duration) and psychological variables (HADS-D) of BC patients and healthy controls. **(A)** Pain characteristics (VAS and pain duration) and HADS-D in BC patients. **(B)** HADS-D were significantly larger in BC patients than that in healthy controls. HADS-D, Hospital Anxiety and Depression Scale-Depression.

Table 1 summarizes sex, age, years of education, pain duration (month), pain intensity [VAS (past) VAS (during scanning)], and depression (HADS-D) in patients with BC and HC (Figure 1A). Sex (*p* > 0.05), age (*t* = 1.153, *p* = 0.256), and educational years (*t* = 0.703, *p* = 0.487) demonstrated a non-significant variation between the two groups. Therefore, the quantified depression condition using HADS-D scales showed significant elevation in the BC group contrasted with the healthy group (*T* = 8.1, *p* < 0.001; Figure 1B). Eight (40% of the total) patients with BC had a HADS-D score ≥ 8.

**Table 1:** Demographic and clinical data of breast cancer (BC) patients and healthy controls (HC) (mean ± SD).

	**BC patients**	**Healthy controls**	***T***	***p***
Female	20	20	-	>0.05
Age (year)	52.25 ± 6.55	49.95 ± 6.06	1.153	0.256
Education (year)	11.10 ± 2.79	11.80 ± 3.47	0.703	0.487
Pain duration (month)	5.05 ± 0.95			
VAS (past)	5.65 ± 1.66			
VAS (scanning)	5.15 ± 1.63			
HADS-D	7.40 ± 3.35	0.75 ± 1.52	8.1	< 0.001

VAS, Visual Analog Scale; HADS-D, Hospital Anxiety and Depression Scale-Depression.

### Associations between pain characters and HADS-D

**Figure 2:**
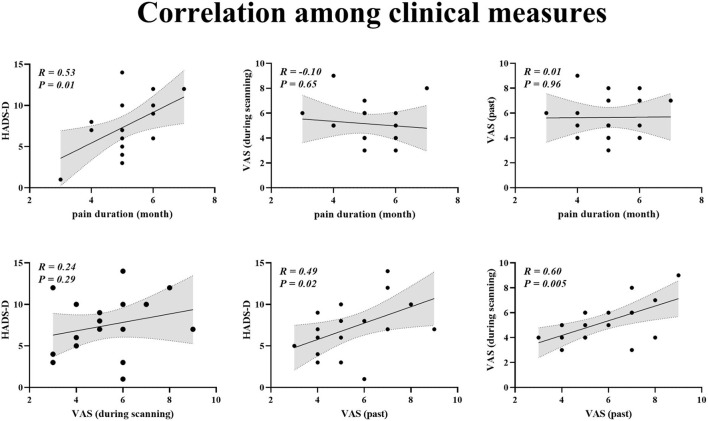
Correlation among clinical measures.

For patients with BC, Pearson correlation analysis demonstrated that pain duration and VAS (past) scores were positively linked to HADS-D scores (pain duration vs. HADS-D: *r* = 0.53, *p* = 0.01; VAS (past) vs. HADS-D: *r* = 0.49, *p* = 0.02; Figure 2). Furthermore, VAS (past) was positively related to VAS (scanning) (*r* = 0.60, *p* = 0.005; Figure 2).

### Seed-based FC

**Figure 3:**
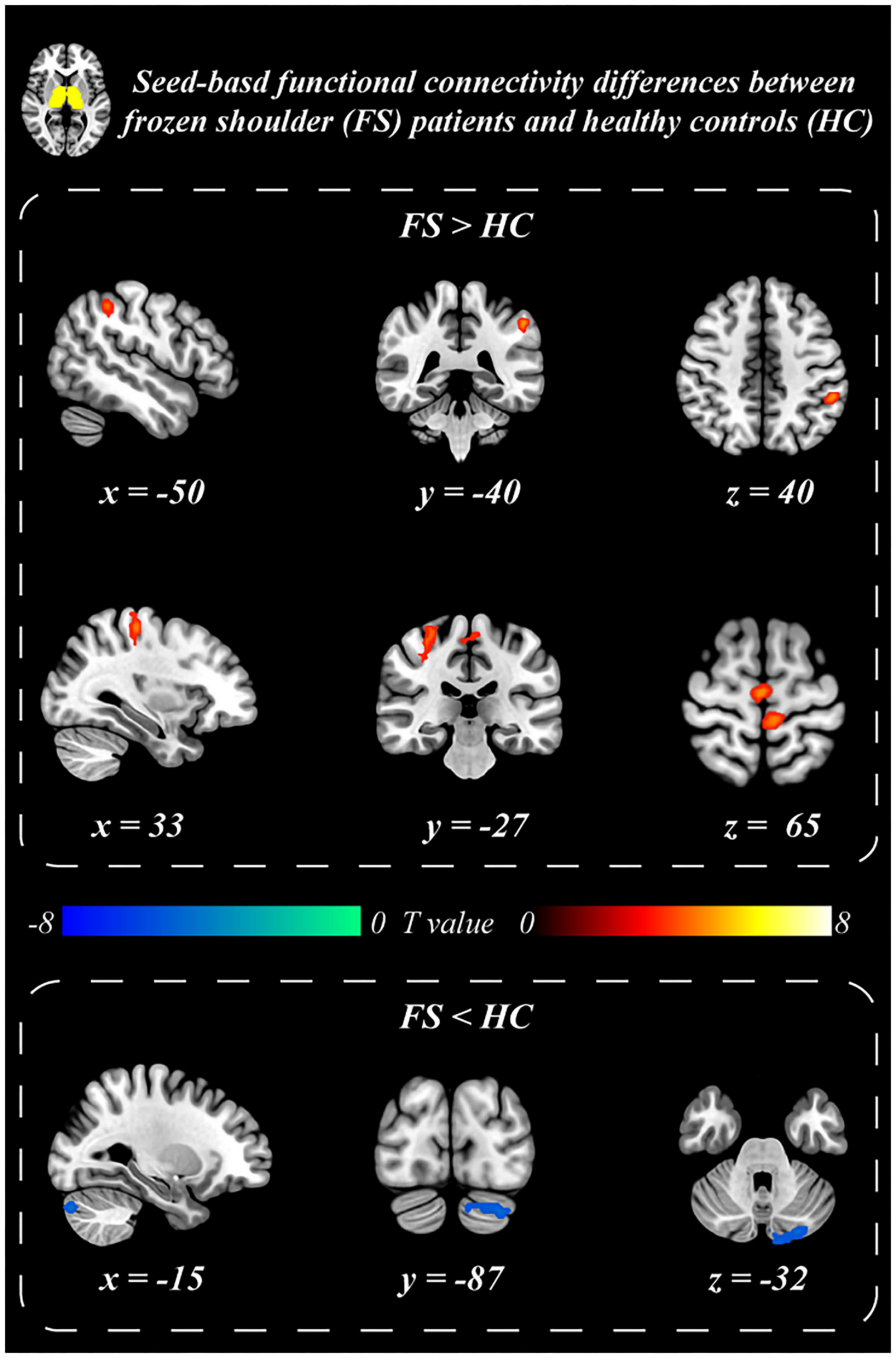
Seed-based functional connectivity differences between BC patients and healthy controls. Thalamus exhibited stronger resting-state functional connectivity with postcentral gyrus (SI) and inferior parietal lobule (IPL), and weaker functional connectivity with the Cerebellum in BC patients than that in the healthy controls.

**Figure 4:**
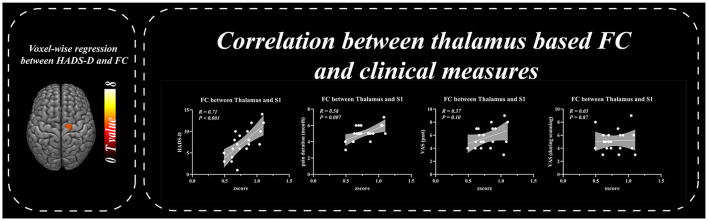
Correlation between thalamus based functional connectivity and clinical measures. Resting-state functional connectivity between thalamus and SI was positively correlated with HADS-D and pain duration in BC patients.

Patients with BC displayed significantly stronger FC with the primary SI and inferior parietal lobule (IPL) than healthy controls when the thalamus was set as the seed *p* ≤ 0.001 (familywise error correction, corrected *p* ≤ 0.05 at cluster level; Table 2, Figure 3). FC between the thalamus and cerebellum of patients with BC was weaker than that of healthy controls *p* ≤ 0.001 (familywise error correction, corrected *p* ≤ 0.05 at cluster level; Table 2, Figure 3). Importantly, HADS-D scales and pain duration were positively correlated with the FC between the thalamus and SI (HADS-D vs. FC: *r* = 0.71, *p* < 0.001; pain duration vs. FC: *r* = 0.58, *p* = 0.007; Figure 4). We identified a non-significant association between thalamus-SI FC and HADS-D in healthy controls. Using PAG as the seed region showed a non-significant variation in FC between the two groups.

**Table 2:** Clusters that exhibited significant seed-based (thalamus) resting-state functional connectivity differences between BC patients and healthy controls.

**Area**	**Side**	**Peak MNI coordinates (x, y, z)**			**Cluster size**	***T*-value**
IPL	L	−50	−40	44	30	4.88
SI	R	33	−27	65	46	4.56
Cerebellum	R	−17	−84	−33	63	−5.03

BC, breast cancer; IPL, inferior parietal lobule; SI, postcentral gyrus.

### Mediation analysis

**Figure 5:**
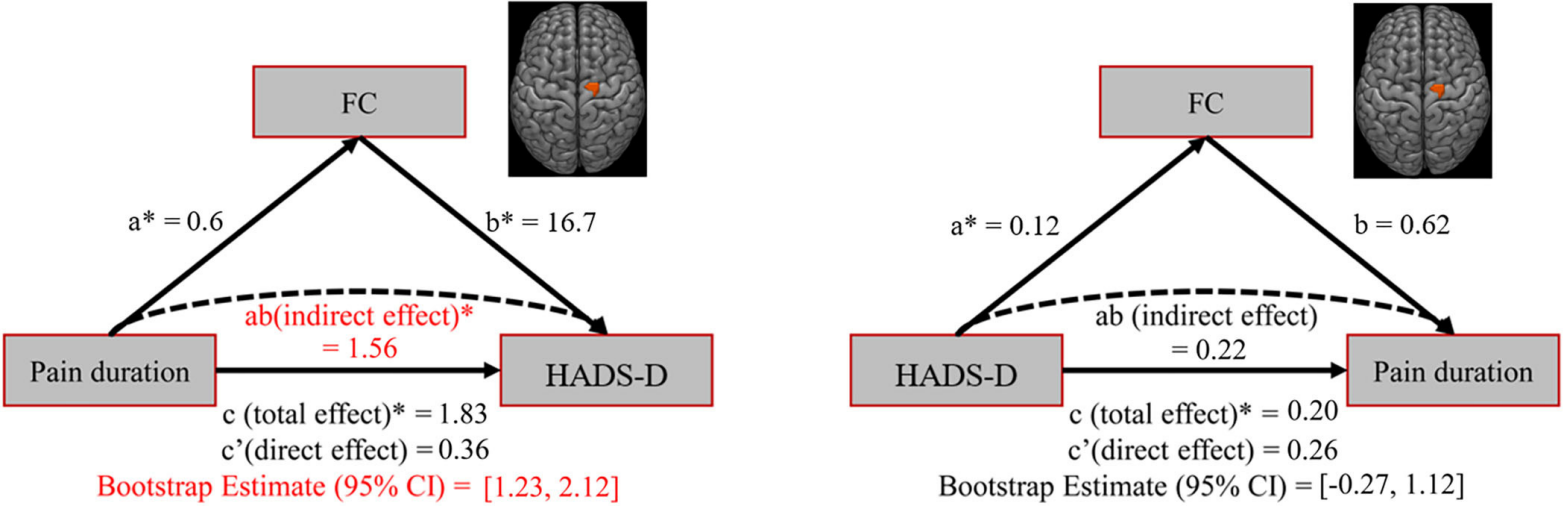
Mediation analysis. **(Left)** The effect of pain duration on HADS-D was mediated by the Thalamus-SI functional connectivity. Path c is the total effect of pain duration on HADS-D; path c' is the direct effect of pain duration on HADS-D after controlling for the Thalamus-SI functional connectivity; the product of a and b (ab) is the indirect effect of pain duration through the Thalamus-SI functional connectivity on HADS-D. **(Right)** The effect of HADS-D on pain duration was not mediated by the Thalamus-SI functional connectivity. Path c is the total effect of HADS-D on pain duration; path c' is the direct effect of HADS-D on pain duration after controlling for the Thalamus-SI functional connectivity; the product of a and b (ab) is the indirect effect of HADS-D through the Thalamus-SI functional connectivity on pain duration. HADS-D, Hospital Anxiety and Depression Scale-Depression. *, *p* < 0.05.

The thalamus-SI FC mediated the pain duration effect on HADS-D (direct effect = 0.36; indirect effect = 1.56, *p* < 0.05; 95% CI: [1.23, 2.12], Figure 5, left panel). In contrast, the impact of HADS-D on pain duration showed no mediation role by the thalamus-SI FC (direct effect = 0.26; indirect effect = 0.22; 95% CI: [−0.27, 1.12], Figure 5, right panel).

### Validation analysis

Our results from validation analyses were consistent with our previous results above. Contrasted to the control group, the BC group exhibited raised FC between the thalamus as well as SI, correlating with the HADS-D score and pain duration. The thalamus-SI FC mediated the impact of pain duration on depression. The above results are presented in the Supplementary material.

## Discussion

Herein, we acquired three primary findings: (1) 40% of patients with BC with CNP had depression, and the HADS-D score was significantly linked to pain duration and VAS (past). (2) Contrasted to the control group, the BC with CNP group exhibited higher FC between the thalamus as well as SI, correlating with the HADS-D score and pain duration. (3) The thalamus-SI FC mediated the impact of pain duration on depression.

Breast cancer is the highest frequent malignancy in women globally and accounts for 25% of female cancer cases (35). CNP and depression are the most common comorbidities in patients with BC, significantly reducing their quality of life and ability to return to society (7). Herein, the incidence of depression in patients with BC was 40%, similar to the reported incidence by previous studies. We also found that depression (HADS-D) was positively correlated with pain duration and VAS (past), consistent with previous studies (15, 16). However, previous studies focused only on this phenomenon and unillustrated their internal connection. Furthermore, we investigated the association between depression and pain in BC patients with CNP through brain function mechanisms.

The neuroimaging study of chronic pain has always focused on the study of pain modulation effects, including pain-associated brain areas and their pain regulation (36, 37). Since the thalamus and PAG serve as the main nodes of the ascending and descending pain regulation pathways, we performed FC analysis using them as seeds. The primary outcome of the fMRI analysis is that the FC between the thalamus and SI and IPL were significantly increased contrasted to the control group. The FC with cerebellum was significantly decreased. The thalamus is the key node of the ascending pain regulation mechanism, transmitting the afferent nociceptive signal from the peripheral receptor to the pain-associated brain region (38). The thalamus plays a crucial role in initiating and maintaining neuropathic pain, like trigeminal neuralgia (39) and chronic back pain (19), and disruption of the thalamocortical network may act as a potential neurobiological indicator of chronic pain (40–43).

Herein, the raised FC of thalamus-SI showed probable function impairments in the ascending pain regulation mechanism in CNP-affected patients with BC. The overloaded input of spontaneous pain in the thalamocortical network might strengthen the association between the thalamus and SI (44), representing that patients with BC were in chronic pain. Moreover, IPL, as a critical component of the default mode network (DMN), is implicated in the brain modification of chronic pain, such as chronic jaw pain (45), chronic musculoskeletal pain (46), and low-back-related leg pain (47). We found that the FC of IPL and the thalamus in the BC group was elevated compared to the control group, suggesting that the sensory monitoring activity can be affected in BC patients with CNP (48, 49).

As the main node in descending pain regulation, PAG is involved in many chronic pain disorders (50–53). Unfortunately, our study undetected changes in the FC of PAG with other brain regions compared with healthy control; the insufficient sample size may be the cause.

We further found that HADS-D and pain duration positively linked to FC between the thalamus and SI through linear regression analysis. This indicated that the malfunction of ascending pain regulation system was strongly linked to CNP and depression. We agreed with prior studies that the thalamus increased resting-state FC with the SI when the intensity and duration of pain increased (22). To explore the relationship among the factors, we performed a mediation analysis of pain duration, HADS-D, and thalamic-SI FC. Moreover, mediation analyses demonstrated that the thalamus-SI FC mediated the impact of pain duration on HADS-D. In contrast, the thalamus-SI FC unmediated the impact of HADS-D on pain duration. A reciprocal correlation existed between pain and depression in patients with chronic pain (32). Chronic pain in patients with BC is a good predictor of depression (54). However, they unexplained the exact mechanism of this phenomenon. Our study explained this phenomenon in terms of brain neural mechanisms, deepening the understanding of CNP and depression in patients with BC.

Accordingly, the present results demonstrated that patients with BC had impairment in ascending pain modulation pathways; pain duration can cause depression through the thalamus-SI connection, which would suggest that even if the relationship between pain and depression is reciprocal, causative effects on one another could be achieved through different neural systems. Indeed, the detailed neural mechanisms responsible for the causative effects need further investigation.

## Conclusion

The ascending pain regulation mechanisms exhibited deficits strongly linked to chronic pain and depression in patients with BC. This research boosted our knowledge of the pathophysiology of CNP in BC, helping to determine the optimal therapy for the patients. The clinical treatment of early pain in patients with BC should be emphasized to prevent the development of CNP. If the patient has developed CNP, paying attention to the depression and dealing with it in time while treating the pain is necessary.

## Limitations and implications

This study has some drawbacks. First, our investigation of the acute association between clinical symptoms and subcortical/cortical dysfunctions was hampered by the limited sample size of individuals with various disease severity and duration. Second, the thalamus has heterogeneous subregions, limiting the illustration of other detailed mechanisms. Third, this study is a complete analysis of the FC method in rs-fMRI, excluding other indicators (amplitude of low-frequency fluctuation and regional homogeneity); we will do further research. Accordingly, we unselected the fMRI data of BC patients with CNP after medication and unexplored the functional differences of the brain after medication. In the future, we will use fs-fMRI to observe brain function changes after medication. Finally, we unexplored peripheral nerve conduction, which may require further clarification using fMRI of the spinal cord.

## Data availability statement

The raw data supporting the conclusions of this article will be made available by the authors, without undue reservation.

## Ethics statement

The studies involving human participants were reviewed and approved by Institutional Review Board of Affiliated Hospital of North China University of Science and Technology. The patients/participants provided their written informed consent to participate in this study.

## Author contributions

All authors listed have made a substantial, direct, and intellectual contribution to the work and approved it for publication.
