# Supplementary material for: Deficits in ascending pain modulation pathways in breast cancer survivors with chronic neuropathic pain: A resting-state fMRI study
Source: Front Neurol. 2022 Dec 8;13:959122. doi: 10.3389/fneur.2022.959122 (PMC9772282; doi:10.3389/fneur.2022.959122)
Supplement: Supplementary file 1 [file Data_Sheet_1.docx]

**Sup-Results**

**Seed-based functional connectivity**

Compared with healthy controls, thalamus exhibited significantly stronger FC with the primary somatosensory cortices (SI), inferior parietal lobule (IPL) and supplementary motor area (SMA) in BC patients (p ≤ 0.001 (familywise error correction, corrected p ≤ 0.05 at cluster level; Sup-Table 1, Sup-Figure 1). The functional connection between the thalamus and cerebellum of BC patients is weaker than that of healthy controls (p ≤ 0.001 (familywise error correction, corrected p ≤ 0.05 at cluster level; Sup-Table 1, Sup-Figure 1). Importantly, HADS-D scales, pain duration and VAS (past) were positively correlated with the FC between thalamus and SI (HADS-D vs FC: *r* = 0.89, *p* < 0.001; pain duration vs FC: *r* = 0.51, *p* = 0.02; VAS (past) vs FC: *r* = 0.61, *p* = 0.005; Sup-Figure 2). When PAG is used as the seed region, there is no significant difference in FC between BC patients and healthy controls.

**Mediation analysis**

The effect of pain duration on HADS-D was mediated by the thalamus-SI FC (direct effect = 0.40; indirect effect = 1.51, *p* < 0.05; 95% confidence interval: [0.61,2.38], Sup-Figure 3, left panel). In contrast, the effect of HADS-D on pain duration was not mediated by the Thalamus-SI FC (direct effect = 0.13; indirect effect = 0.24, 95% confidence interval: [- 0.32, 0.24], Sup-Figure 3, right panel).

**Sup-Figure 1**


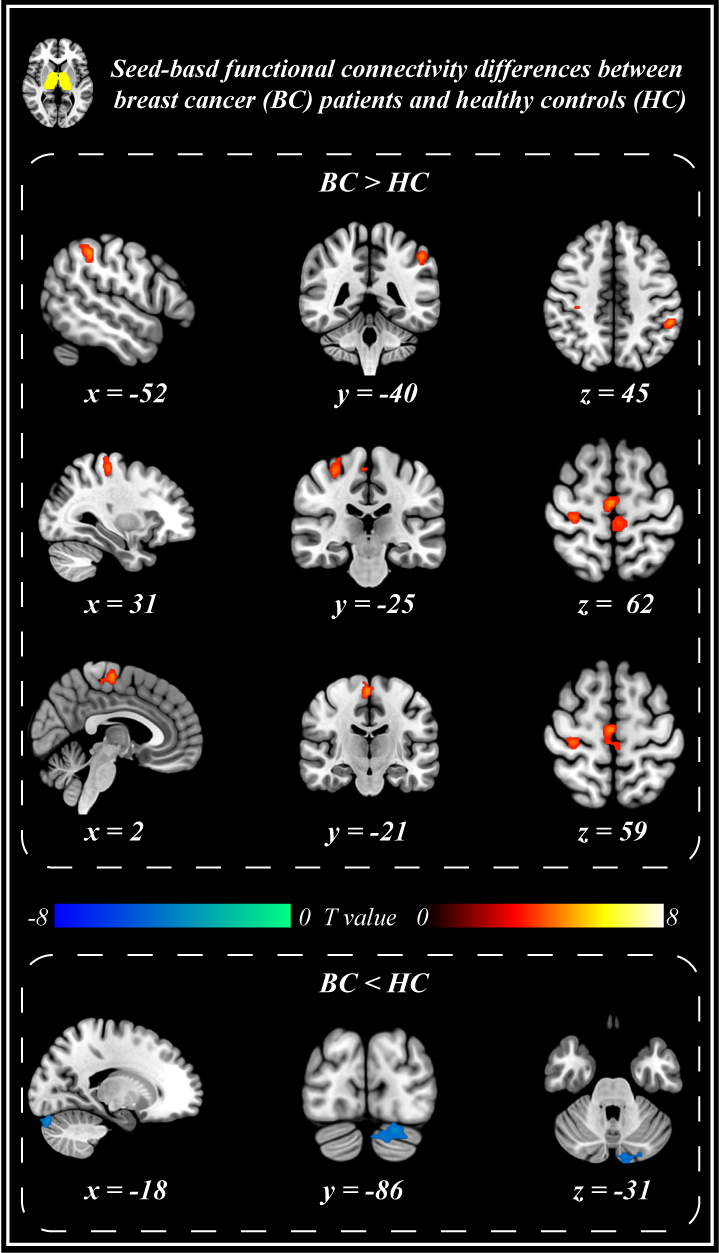


Seed-based functional connectivity differences between BC patients and healthy controls.

Thalamus exhibited stronger resting-state functional connectivity with the SI, inferior parietal lobule and supplementary motor area, and weaker functional connectivity with the Cerebellum in BC patients than that in the healthy controls.

**Sup-Figure 2**


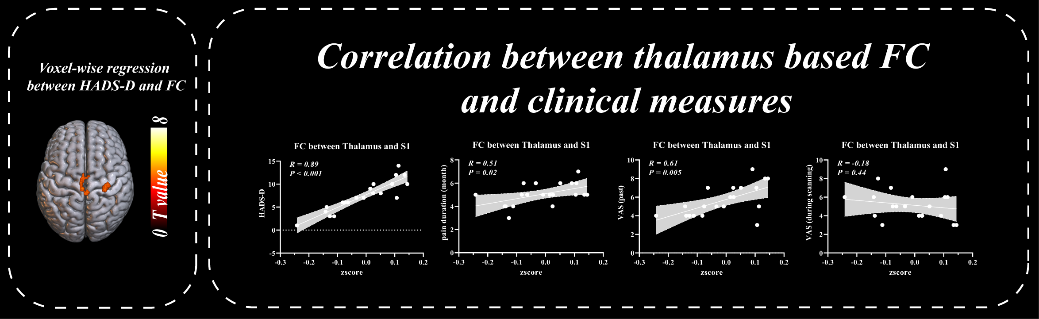


Left panel. Voxel-wise regression between thalamus based functional connectivity and clinical measures. Right panel. Resting-state functional connectivity between thalamus and SI was positively correlated with HADS-D, pain duration in BC patients.

**Sup-Figure 3**


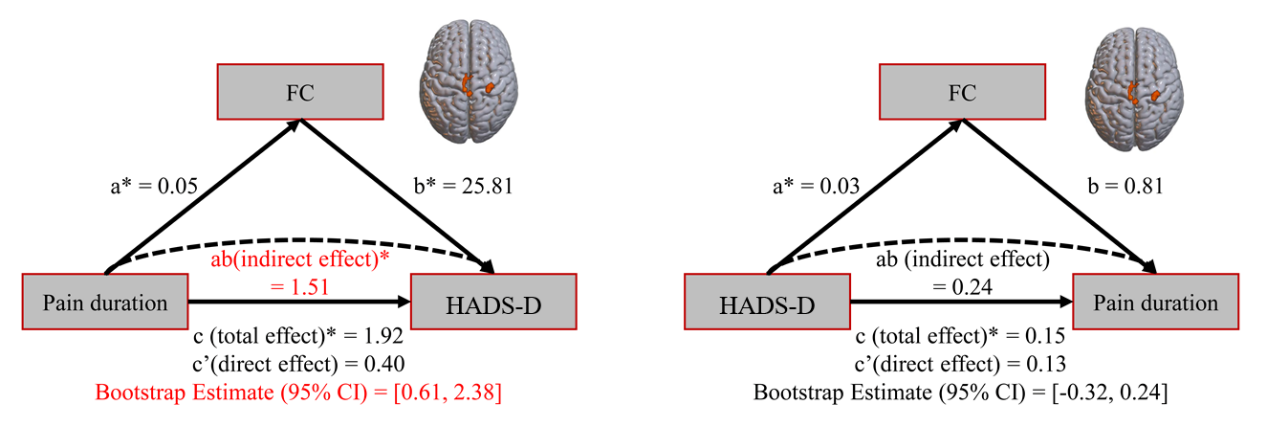


Left panel: The effect of pain duration on HADS-D was mediated by the Thalamus-SI functional connectivity. Path c is the total effect of pain duration on HADS-D; path c’ is the direct effect of pain duration on HADS-D after controlling for the Thalamus-SI functional connectivity; the product of a and b (ab) is the indirect effect of pain duration through the Thalamus-SI functional connectivity on HADS-D.

Right panel: The effect of HADS-D on pain duration was not mediated by the Thalamus-SI functional connectivity. Path c is the total effect of HADS-D on pain duration; path c’ is the direct effect of HADS-D on pain duration after controlling for the Thalamus-SI functional connectivity; the product of a and b (ab) is the indirect effect of HADS-D through the Thalamus-SI functional connectivity on pain duration.

HADS-D: Hospital Anxiety and Depression Scale-Depression. ∗: p < 0.05.

**Sup-Table 1.** Clusters that exhibited significant seed-based (thalamus) resting-state functional connectivity differences between BC patients and healthy controls.

| Area | Side | Peak MNI coordinates  (x, y, z) | | | Cluster size | *T* value |
| --- | --- | --- | --- | --- | --- | --- |
| IPL | L | -52 | -40 | 45 | 33 | 4.91 |
| SI | R | 31 | -25 | 62 | 51 | 4.69 |
| SMA | R | 2 | -21 | 59 | 71 | 4.85 |
| Cerebellum | R | -18 | -86 | -31 | 60 | -4.53 |

Abbreviations: BC, breast cancer; IPL, inferior parietal lobule; SI, postcentral gyrus; SMA, supplementary motor area.
